# Food Allergens of Plant Origin

**DOI:** 10.3390/foods12112232

**Published:** 2023-06-01

**Authors:** Yuzhu Zhang, Huilian Che, Caiming Li, Tengchuan Jin

**Affiliations:** 1 US Department of Agriculture, Agricultural Research Service, Pacific West Area, Western Regional Research Center, 800 Buchanan Street, Albany, CA 94710, USA; licaiming2009@126.com; 2 Key Laboratory of Precision Nutrition and Food Quality, Key Laboratory of Functional Dairy, Ministry of Education, College of Food Science and Nutritional Engineering, China Agricultural University, Beijing 100083, China; chehuilian@cau.edu.cn; 3 School of Food Science and Technology, Jiangnan University, Wuxi 214122, China; 4 Collaborative Innovation Center of Food Safety and Quality Control, Jiangnan University, Wuxi 214122, China; 5 Department of Obstetrics and Gynecology, The First Affiliated Hospital of USTC, Division of Life Sciences and Medicine, University of Science and Technology of China, Hefei 230001, China; jint@ustc.edu.cn

**Keywords:** allergenicity, epitopes, vicilin leader peptide cC3C, plant allergen structure

## Abstract

This review presents an update on the physical, chemical, and biological properties of food allergens in plant sources, focusing on the few protein families that contribute to multiple food allergens from different species and protein families recently found to contain food allergens. The structures and structural components of the food allergens in the allergen families may provide further directions for discovering new food allergens. Answers as to what makes some food proteins allergens are still elusive. Factors to be considered in mitigating food allergens include the abundance of the protein in a food, the property of short stretches of the sequence of the protein that may constitute linear IgE binding epitopes, the structural properties of the protein, its stability to heat and digestion, the food matrix the protein is in, and the antimicrobial activity to the microbial flora of the human gastrointestinal tract. Additionally, recent data suggest that widely used techniques for mapping linear IgE binding epitopes need to be improved by incorporating positive controls, and methodologies for mapping conformational IgE binding epitopes need to be developed.

## 1. Introduction

Food allergies are adverse immune responses to foods. The symptoms of a food allergy range from mild hives and itching to life-threatening anaphylaxis. In the US, it was estimated that up to 26 million adults [1] and 6 million children [2] have food allergies. Depending on the methods of studies, the sub-population suffering from food allergies in Europe was estimated between 0.8% (by positive food challenge) to 19.9% (by a survey of self-reported food allergies) [3], and the overall food allergy prevalence in Asia is comparable to that in the West [4]. Food allergies are among the top causes of anaphylaxis that lead to children’s visits to emergency departments in the United States [5]. In addition, the situation is worsening as food allergy prevalence has increased in the past few decades [2,6,7,8]. Most food allergy reactions are the immediate type that happens within hours of food intake. They are reactions to proteins (except for a small number of cases, see below) mediated by immunoglobulin E (IgE) antibodies. Food allergy reactions happen because the immune system has previously, for unknown reasons, mistaken a food protein as a dangerous invader, switched the class of T helper cells that determines whether the B cells produce IgG or IgE, and developed IgE antibodies against the protein in a so-called sensitization stage. Sensitization to food can happen in an individual when that person consumes it for the first time. It can also occur in people even though they have been eating the food safely for years to decades. In the sensitization stage, abnormal immune responses promote the class switching of B-cells to produce IgE antibodies specific to a food protein and clonal expansion of naive and IgE^+^ memory B-cell populations [9]. IgE molecules bind to the surface of mast cells and basophils through their association with the high-affinity IgE receptor FcεRI. Subsequent consumption of the food by the patient leads to allergen cross-linking of the IgE antibodies, which, in turn, results in the initiation of allergic reactions via signaling through the high-affinity receptor for the Fc region of immunoglobulin E (IgE) or FcεRI [10,11]. Extensive research on food allergies has been conducted in recent years. Most of these efforts involved studying the genetic, environmental, and other factors that cause a sub-population to develop a food allergy [12]. In comparison, less research has been devoted to studies on the offending allergens. Nevertheless, research interest in allergic food has increased with the upsurge in the prevalence of food allergies, and more and more allergens have been identified, especially in recent years. Here, we present the result of a comprehensive review and analysis of the chemistry of well-established and recently defined food allergens and their families.

## 2. Food Allergens

Allergens are given names by the Allergen Nomenclature Sub-committee, which operates under the auspices of the World Health Organization (WHO) and the International Union of Immunological Societies (IUIS) [13]. The approved name contains three parts with separations by a space, three letters for the genus being the first part, one letter for the species as the second part, and an Arabic number. The letters are those at the beginning of the genus and species names and the Arabic number indicates the order of the identification of the allergens in that species [13]. The fourth letter of the genus and/or the second letter of the species is included when necessary to remove ambiguity. The WHO/IUIS Allergen Nomenclature Sub-committee also maintains a database of allergens with designated names. This database currently contains 248 food allergens of plant sources from 76 species.

In the early days of plant protein studies, they were classified based on their solubility and extractability in a series of solvent extractions and they were grouped into four groups (albumins, globulins, prolamins, and glutelins) [14]. With the advancement of knowledge of the function, biochemical, and molecular properties of proteins, plant proteins can also be classified into three groups based on their functions, structural and metabolic proteins, protective proteins, and storage proteins. Metabolic proteins can be named by their biochemical activity, while storage proteins are generally those without a known cellular activity other than the storage of nitrogen, carbon, and sulfur for the development of the next generation of the plant. Protective proteins are those with a function to defend the plant against pests, microbial pathogens, or environmental stresses. In the field of modern protein research, one of the methods of obtaining valuable information on proteins by analyzing their sequence, structure, and function is the Pfam classification of protein families based on hidden Markov model profiles [15]. At present, proteins are classified into about 19 thousand Pfam signatures [16].

The number of protein families that contain proteins from plant sources that are known to be capable of eliciting allergic responses in atopic individuals is several orders of magnitude lower compared to the total number of Pfam signatures. There are thousands of proteins in a mature plant seed [17,18], but 79% of plant food allergens belong to only 12 protein families. Table 1 listed the protein families that are known to contain more than two food allergens along with the number of known allergens in each of the families. Some of the allergens (e.g., chitinases) include two or more domains that belong to different protein families. In addition to those shown in Table 1, six protein families contain two allergens. Twenty-four allergens of plant sources belong to protein families that contain only one known allergen. Nine allergens do not belong to any of the classified Pfam families, i.e., searching the Pfam database with the allergen sequences using the online search tool at the site of the Pfam database did not find any hit. Note that minimal sequence information was available about four of the allergens in the database.

The protein family with the most food allergens from plant sources is PF00234 (the protease inhibitor/seed storage/LTP family), which has 74 known allergens. This family includes the plant nonspecific lipid transfer proteins (NsLTP), such as NsLTP1 and NsLTP2, the 2S albumin seed storage proteins, trypsin/alpha-amylase inhibitors, and other proteins. The next protein family is the Cupin-1 family, which includes 39 allergens that are in the WHO/IUIS allergen database. They are also seed storage proteins with close to half of the allergens belonging to the 11S legumins and half belonging to the 7S vicilins. Three of these allergens are now renamed as isoforms of other allergens, but their entries in the database stay due to historical reasons and literature references. With 27 food allergens, the profilin family ranked third in containing more food allergens from plant sources. Thus, the top three protein families contain more than half of the known plant food allergens. In addition, numerous other profilins are known to be pollen allergens. This indicates that the biological activities, physical–chemical properties, and conserved structures of the allergens may play a role in determining or promoting their allergenicity. The following describes the leading plant food allergen families:

**Nonspecific lipid transfer proteins.** Nonspecific lipid transfer proteins (NsLTPs) are found in all land plants [19]. They are small proteins with molecular masses of around ten kDa. They were demonstrated in vitro to be able to bind and transport various phospholipids to chloroplasts or mitochondria without specificity [20]. NsLTPs are plant pathogenesis-related proteins known as PR-14, and a number of them have been demonstrated to have antimicrobial activities including NsLTPs in wheat (*Triticum aestivum* L.) [21,22] and mung bean (*Vigna radiata* L. *R. Wilczek*) [23]. NsLTPs can be identified by an eight-cysteine residue motif (8CM). Based on the number of residues separating one cysteine from the next and the conservation of residue types at specific positions of the flanking sequences, the NsLTPs can be divided into two types. The 8CM motif for NsLTP1 is CX_2_VX_5–7_C[VLI]XY[LAV]X_8–13_CCXGX_12_DX[QKR]X_2_CXCX_16–21_PX_2_CX_13–15_C, and that for NsLTP2 is CX_4_LX_2_CX_9–11_P[ST]X_2_CCX_5_QX_2–4_C[LF]CX_2_[ALI]X[DN]PX_10–12_[KR]X_4–5_CX_3–4_PX_0–2_C [24], where X with a subscript number represents the number of non-conserved amino acids residues and allowed residue variation at a single position is placed in a square bracket. Thus, C1X_7–10_C2X_12–17_C3C4X_8–19_C5XC6X_19–24_C7X_4–15_C8 can be used to describe the 8CM of the plant NsLTPs, where the Cys residues are numbered from 1 to 8. The functions of NsLTPs are not well understood, but their expression levels are known to be high in most tissues, indicating that they may be essential for the reproduction and survival of plants. Four NsLTP2s and 38 NsLTP1s are known to be food allergens. Known NsLTP food allergens from the major allergen sources recognized by US Food and Drug Administration (FDA) include peanut (*Arachis hypogaea* L.) allergen Ara h 9 [25] and Ara h 17 [26], almond (*Prunus dulcis* (Mill.) D.A.Webb) allergen Pru du 3 [26], chestnut (*Castanea sativa* Mill.) allergen Cas s 8 [27], hazelnut (*Corylus avellana* L.) allergen Cor a 8 [28], walnut (*Juglans regia* L.) allergen Jug r 3 [29], and wheat allergen Tri a 14 [30]. Furthermore, the NsLTPs from many plants not used for food are known to be pollen allergens.

**Figure 1:**
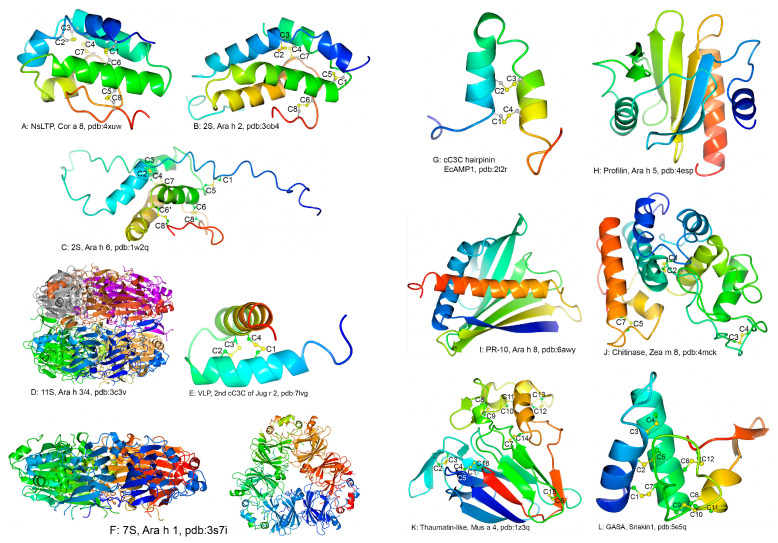
Structures of representative members of protein families that contain the majority of the known food allergens of plant origin. The name of the allergen or protein and the protein family/subfamily is indicated below every structure following the (**A**–**L**) sequence label of the individual panels. The coordinates of the structures were downloaded from the WorldWide Protein Data Bank, and the graphics displays were generated using the CCP4MG program. The PDB codes for the structures are included in the figure labels along with the names of the allergens. Each structure is shown as a ribbon diagram with a blend-through coloring scheme displaying the *N*-terminal blue and the *C*-terminal red, except for the multimeric allergens Ara h 1 and Ara h 3 where the monomers were blended through different color ranges. Two panels of Ara h 1 are presented, with the right panel being the left panel rotated about a horizontal axis parallel to the paper pointing to the right. The side chains of cysteines that are involved in disulfide bonds are shown as ball-and-stick. Cysteines that are conserved in well-defined sequence motifs are labeled with their numbering in the motifs (see text).

The crystal structure Cor a 8 [31] was the first structure reported for an NsLTP food allergen from the major allergen sources, though that of wheat allergen Tri a 14 [32] and the solution structure of Tri a 14 [33] were reported many years ago before it was identified as a food allergen. As shown in Figure 1A, the cysteines in the 8CM of Cor a 8 form four disulfide bonds. Protein structures were generated with the CCP4MG program [34]. The structures of many other NsLTP1 food and pollen allergens are also available. The conservation of these disulfide bond connectivities (between C1–C6, C2–C3, C4–C7, and C5–C8) in NsLTP1s maintains the tertiary contacts of the secondary structural elements and ensures a stable hydrophobic cavity for lipid binding [35]. Moreover, the structure of rice (*Oryza sativa* L.) NsLTP2 was determined by NMR, which showed an overall structure similar to that of an NsLTP1. The disulfide bond connectivities in NsLTP2 (C1–C5, C2–C3, C4–C7, and C6–C8) are different from those in NsLTP1 [36].

**2S albumins.** Plant proteins coagulable by heat and soluble in water were called albumins in the early 20th century for their properties that resembled hen egg albumin [14]. The 2S albumins migrated with a 2S sedimentation coefficient during sucrose gradient centrifugation [37]. The 2S albumins also contain an 8CM similar to that of the NsLTPs but with longer sequences separating C2 and C3 and C6 and C7. Seed storage proteins are believed to accumulate in developing seeds to act as a nitrogen reserve for germination [38,39]. The 2S albumins were considered to be a major group of storage proteins in many dicotyledonous plant species [40] that also play a role in providing sulfur reserve in the seed [37]. The 2S albumins have also been suggested to have antimicrobial activities [41,42,43]. Known 2S food allergens from the major allergen sources recognized by FDA include peanut allergen Ara h 2 [44], soybean (*Glycine max*) allergen Gly m 8 [45], Brazil nut (*Bertholletia excelsa* Silva Manso) allergen Ber e 1 [46], cashew (*Anacardium occidentale* L.) allergen Ana o 3 [47], hazelnut allergen Cor a 14 [48], pecan (*Carya illinoinensis* (Wangenh.) K.Koch) allergen Car i 1 [49], pistachio (*Pistacia vera* L.) allergen Pis v 1 [50], Stone pine (*Pinus pinea* L.) allergen Pin p 1 [51], sesame (*Sesamum indicum* L.) allergens Ses i 1 [52] and Ses i 2 [53], and Black walnut (*Juglans nigra* L.) allergens Jug n 1 and walnut allergen Jug r 1 [54].

The structures of many 2S albumins including food allergens in castor beans (*Ricinus communis* L.) (Ric c 1) [55], rapeseed (*Brassica napus* L.) (Bra n 1) [56], and Brazil nuts (Ber e 1) [57] have been reported. The first structure reported for a 2S albumin allergen from the major allergen sources is that of Ara h 6, which was determined by NMR using recombinantly expressed Ara h 6 with uniform ^15^N and ^13^C labeling [58]. Three peanut 2S albumins have been identified as food allergens. They are Ara h 2, Ara h 6, and Ara h 7. The structure of Ara h 2 was also reported (Figure 1B). It was determined by X-ray crystallography using recombinantly expressed Ara h 2 with a maltose-binding protein fused to the *N*-terminal to enhance its solubility and aid its crystallization [59]. These 2S albumins have the same disulfide bond connections as NsLTP2. However, Ara h 6 has an additional disulfide bond, which is formed by an extra cysteine between C6 and C7 (C6′) and another cysteine residue after C8 (C8′), as shown by one of the models of its structures determined by NMR (Figure 1C).

**11S legumins.** Both the 11S and the 7S seed storage proteins belong to the cupin superfamily, which was initially recognized based on a 50% sequence identity between the wheat protein germin and a slime mold (*Physarum polycephalum)* protein spherulin [60]. Germin is an unusually thermostable protein produced during the early phase of germination in wheat embryos. The sequence similarity was then extended to a group of germin-like proteins and globulin storage proteins. Globulins were classified as those soluble in dilute salt solution but insoluble in water [14]. After structural information on canavalin [61] and phaseolin [62] became available, sequence alignment revealed a much larger group of proteins in this superfamily, and the family was given the name cupin [63] (from the Latin term ‘*cupa*’ which means small barrel). The cupin superfamily contains monocupin, bicupins, and multicupins. It is known to be one of the most functionally diverse protein superfamilies [64] including various proteins with enzymatic functions, non-enzymatic transcription factors, and the 11S and 7S seed storage proteins.

The signature of the cupins includes two sequence motifs separated by an inter-motif sequence with variable length (from 11 amino acids to over a hundred residues). The first motif was defined as GX_5_HXHX_3–4_EX_6_G, and the second motif was characterized as GX_5_PXGX_2_HX_3_N. The two histidines and the glutamate in motif 1 and the histidine in motif 2 may act as ligands to bind metal ions. In many cupins with enzymatic activity, a metal ion is part of the active site [65]. However, the motifs are now known to tolerate variations and not all cupins have a metal ligand [64]. Nevertheless, residues other than those specified above can also provide metal ligand coordination [66].

The 11S globulins are the most widespread among seed storage protein groups. They are present in monocot and eudicot seeds, as well as in conifers and other gymnosperms. They are particularly abundant in legume seeds and are often called legumins. Typical legumins have molecular weights (MW) of about 300–450 kD and consist of six subunits of about 60 kD. These subunits are the products of a multigene family. Each subunit is post-translationally processed to give rise to an acidic (MW about 40 kD) chain and a basic (~20 kD) chain [67]. The acidic and the basic chains are linked by a single disulfide bond. The 11S globulins are rarely, if ever, glycosylated. This family of proteins accounts for many of the known major food allergens from the FDA-recognized major allergen sources including peanut allergen Ara h 3 [68], soybean allergen Gly m 6 [69], almond allergen Pru du 6 [70], Brazil nut allergen Ber e 2, cashew allergen Ana o 2 [71], hazelnut allergen Cor a 9 [72], macadamia (*Macadamia integrifolia* Maiden and Betche) allergen Mac i 2 [73,74], pecan allergen Car i 4 [75], pistachio allergen Pis v 2 [50], sesame allergens Ses i 6 and Ses i 7 [76], and walnut allergens Jug n 4 [77] and Jug r 4 [78].

The first structure reported for a legumin food allergen from the FDA-recognized major food sources is that of Gly m 6, which was determined before it was designated as a food allergen [13,79]. The 11S seed storage proteins in many species have more than one type of subunit and five for Gly m 6 [79]. Mature 11S proteins are hexamers that can be composed of different subunits, making it problematic for crystallization. The crystal structure of Gly m 6 was determined by purifying the protein from genetically modified soybeans with four of the subunits of the 11S protein deleted. The peanut 11S food allergen is also known to be coded by at least five different genes [80]. Nevertheless, the population of the mature protein that is composed of the translation from a single gene may be high, and the crystallization of Ara h 3 from wild-type peanuts was successful [81]. The first crystal structure of a peanut allergen was that of Ara h 3 (Figure 1D) [82]. The structure of another 11S allergen from the FDA-recognized major food sources, Pru du 6, was also determined [83]. Generally, the 11S allergens are a dimer of trimers. While the doughnut-shaped trimer was made up of three subunits by head-to-tail associations, the back-to-back binding of the trimers forms the hexameric structure of the native molecule. The dimerization of the trimers buries the *N*-terminus of the basic subdomain which was generated as a result of the cleavage at a conserved peptidase recognition site. The *C*-terminal of the acidic domain, however, moved away before the trimer–trimer interface to facilitate the packing of the mature hexamer, making it impossible to express the 11S allergen recombinantly with most of the commonly used strategies [83]. The structure of an 11S putative allergens purified from coconut was also determined recently [84,85].

**7S vicilins.** The 7S globulins are called vicilins, and they are also present in flowering plants and other spermatophytes. Vicilins are trimeric proteins of MW of ~150–190 kD, with a typical subunit MW of ~50 kD. No disulfide bond was found in vicilins, but proteolytic processing and glycosylation may occur [86,87]. Thus, the subunit structure of vicilins revealed by SDS-PAGE is similar in the absence or presence of reducing agents. Vicilins also account for many known major food allergens from the FDA-recognized major allergen sources including peanut allergen Ara h 1 [88], soybean allergen Gly m 5 [69], almond vicilin [89], cashew allergen Ana o 1 [90], hazelnut allergen Cor a 11 and Cor a 16 [91,92], macadamia allergen Mac i 1 [73], pecan allergen Car i 2 [93], pistachio allergen Pis v 3 [94], Korean pine (*Pinus koraiensis* Siebold and Zucc.) allergen Pin k 2 [95,96], walnut allergens Jug n 2 and Jug r 2 [97], and sesame allergen Ses i 3 [53].

**Figure 2:**
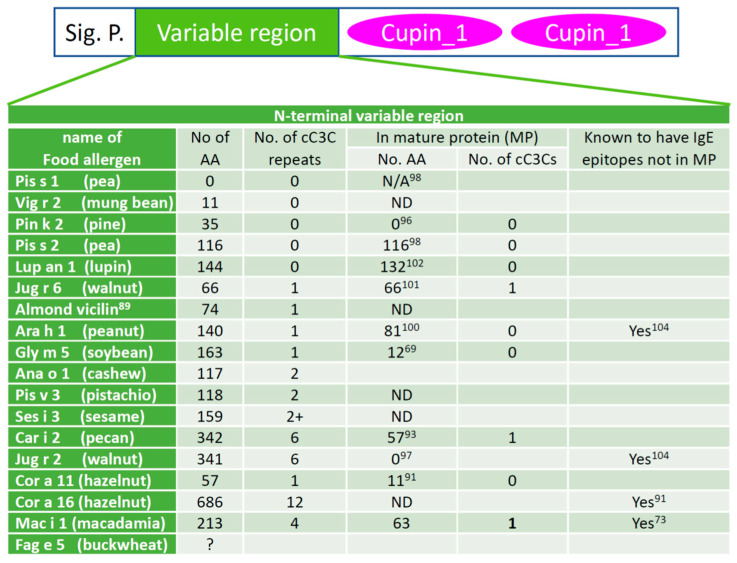
cC3C repeats of the variable leader peptide of known vicilin allergens. The domain structure of vicilin is shown at the top. Several features of the *N*-terminal variable region of vicilin between the signal peptide (Sig. P.) and the *C*-terminal cupin domains are shown below the domain structure. A question mark in the second column indicates the full sequence of the variable region of the allergen is not available. “ND” means no data available, and superscripts indicate the reference number of the cited literature. The variable leader peptides are derived by determining the signal peptides and the *N*-terminal peptides of the natural allergens. The sequences of the allergens were downloaded from the protein database at NCBI (https://www.ncbi.nlm.nih.gov/protein/, accessed on 1 April 2023). The signal peptides were predicted using SignalP 6.0 and the *N*-termini were those reported in the relevant references. The reference numbers are given as superscripts in the table cells. Almond vicilin was reported to be an allergen but does not have an Allergen Nomenclature Subcommittee designated allergen name.

**Vicilin leader peptides.** Vicilins from some species, such as pea (*Pisum sativum* L.) allergen Pis s 1 [98], contain just the di-cupin region and a signal peptide. However, vicilins from other species are known to have a variable region between the signal peptide, which can be predicted [99], and the *C*-terminal di-cupin domains [100,101,102]. This variable leader peptide (VLP) was also called vicilin leader peptide. When they are found in a food independent of the mature vicilin protein with demonstrated allergenicity, they are designated as vicilin iso allergens by the WHO/IUUS Allergen Nomenclature Subcommittee, e.g., Ara h 1.0101 (26–84). In many vicilins, this region can consist of one (as in almond vicilin allergen [89] and peanut allergen Ara h 1 [88]) or more (as in pecan allergen Car i 2 [93]) repeats of a coupled-C3C (cC3C) motif which has a quintet of cysteines arranged as a pair of CX_3_C linked by 8–12 amino acids. Interestingly, none of this variable region, part of it, or the entirety of the region can be found in the native vicilin purified from the seeds, depending on the plant species. The cC3C area of macadamia nut vicilin was reported to have antimicrobial activities [103]. Available data on the cC3C repeat in the variable region of vicilin food allergens are summarized in Figure 2.

The structures of the cC3C of the VLP of Ara h 1, the two individual cC3C repeats of the VLP of Ana o 1 and Pis v 3, and three cC3C repeats of the VLP of Jug r 2 have been determined by NMR [104,105]. The structures of such cC3C are typical disulfide bond stabilized helix hairpins and the best model of the structure bundle of the second cC3C repeat of Jug r 2 is shown in Figure 1E.

The VLP was also referred to as vicilin buried peptide [104,105]. However, none of the known structures of vicilins included the VLP region of proteins. The vicilins used for structure determination were either those purified from seeds without a cC3C repeat in the mature proteins or recombination proteins that lacked a cC3C region. Figure 1F shows the structure of peanut allergen Ara h 1 [106,107]. A vicilin allergen is a doughnut-shaped trimer, similar to the trimers that dimerize to form the hexameric legumin food allergens. The crystal structures of other vicilin food allergens from the FDA-recognized major food sources have also been reported. These include hazelnut allergen Cor a 11 [108], pecan allergen Car i 2 [93], and pine nut allergen Pin k 2 [95]. The structure of one of the vicilin subunits of soybean that is an isoform of Gly m 5 was also determined [109]. The structures determined earlier for the cupin family proteins were also vicilin structures [61,62].

**The cC3C proteins.** The cC3C region defines a separate protein family (PF04702) named vicilin_N in the Pfam classification. However, an almond protein that is made up entirely of cC3C repeats has also been identified as a food allergen (Pru du 8) [110]. Its orthologs can also be found in many other species [110,111]. One class of known antimicrobial peptides is the α-hairpinins, which are known to be produced from multimodular precursor proteins. They contain a single cC3C mofit [112] and assume a helix-loop-helix structure with two stabilizing disulfide bonds, as demonstrated by the structure of EcAMP1 from seeds of barnyard grass (Figure 1G) [113]. Whether this disulfide fortified helix-loop-helix is a building block, and the structure of the PF04702 family of allergens remains to be investigated.

**Profilins.** Profilin is an actin-associated protein that is present in all eukaryotic cells. It was first identified as an allergen in birch pollen [114]. Now it is known to be food allergens in nuts and beans [44,115,116], fruit [117,118,119], and vegetables [120,121]. It has also been identified as pollen allergens from numerous plant sources [122,123]. Thus, it is considered to be a panallergen [123,124] that is often responsible for cross-reactivity [122,124,125]. In the FDA-recognized major allergen sources, known profilin allergens include peanut allergen Ara h 5 [44], Soybean allergen Gly m 3 [116], almond allergen Pru du 4 [126], hazelnut allergen Cor a 2 [127], walnut allergen Jug r 7, and wheat allergen Tri a 12.

The first reported three-dimensional structure of a profilin food allergen is the crystal structure of peanut allergen Ara h 5 (Figure 1H) [128]. Also available is the crystal structure of another profilin food allergen recently determined for muskmelon (*Cucumis melo* L.) allergen Cuc m 2 [129]. In addition, a number of structures of profilin airway and contact allergens have been reported, including those of birch (*Betula verrucosa* Ehrh) allergen Bet v 2 [130], corn (*Zea mays* L.) allergen Zea m 12 and latex (*Hevea brasiliensis* (Willd. ex A.Juss.) Müll.Arg.) pollen allergen Hev b 8 [131]. Many structures of mammalian profilins have been reported, including those of human, mouse, rat, and cattle profilins.

**Pathogenesis related-10 protein.** The plant pathogenesis-related 10 (PR-10) proteins are homologous to one of several families of proteins that are upregulated in response to pathogens (such as viruses, bacteria, and fungi) or induced by a wound or adverse environmental stresses [132,133]. Currently, PR proteins are divided into seventeen classes [134]. PR-10 proteins are not related to other classes of PR proteins, and they are expressed constitutively in many parts of the plant [135]. Members of this class of proteins are known to bind to small hydrophobic compounds and biologically important ligands such as fatty acids, cytokinins, sterols, flavonoids, and emodin [136,137]. They are also known to have antimicrobial activities and are reported to have RNase activities [138,139].

PR-10 from 19 species is known to be food allergens including those from peanut (Ara h 8) [140], soybean (Gly m 4), walnut (Jug r 5), and almond (Pru du 1). PR-10 is also known to be pollen allergens in chestnut (Cas s 1) [141], hazelnuts (Cor a 1) [142], and numerous other species. Thus, PR-10 is also one of the cross-reactive plant allergens considered to be a panallergen [143].

The structures have been determined for numerous PR-10 allergens including many food allergens. From the FDA recognized major food allergen sources, hazelnut allergen Cor a 1 [144], peanut allergen Ara h 8 [145] (Figure 1I), and soybean allergen Gly m 4 [146] have been structurally characterized.

**Oleosin.** Ten known food allergens belong to the oleosin protein family (PF01277). However, they are food allergens from four species; four of them are peanuts allergens, three of them are hazelnut allergens, and two are sesame allergens. The Tartarian buckwheat (*Fagopyrum tataricum* (L.) Gaertn) allergen Fag t 6 is a recent addition to the oleosin allergen family [147]. Oleosins are one of the main classes of proteins associated with plant oil bodies [148]. Oil bodies are natural oil droplets that can be found in yeast, mammalian cells, and many parts of a plant including leaves, flowers, pollens, and seeds [149]. The oil bodies of plant seeds contain mainly triacylglycerols that are enclosed by a monolayer of phospholipids and associated membrane proteins [150]. Oil bodies are dynamic cellular organelles that function as storage structures, protein trafficking, and lipid catabolism [151]. Oil body-associated proteins are involved in diverse activities, including freeze tolerance and defense against fungal infection [152]. Oleosins were also reported to have acyltransferase and phospholipase activities [153]. To date, no structure of an oleosin food allergen has been determined, but it was predicted to have a central conserved hydrophobic domain that anchors to the phospholipid monolayer flanked by two none conserved amphipathic domains that reside on the surface of oil bodies [154].

**Chitinases.** Seven of the known food allergens contain a domain belonging to the protein family of chitin recognition protein (PF00187) which is one of the numerous subgroups of carbohydrate-binding modules. Four of the other allergens are pathogenesis-related proteins known as PR-3. They are class I and class IV chitinases that also have a domain belonging to the glycoside hydrolase family 19 (PF00182). Chitin, one of the most abundant polysaccharides on the planet, is a β-(1,4)-linked homopolymer of *N*-acetylglucosamine. Chitin is an indispensable element of the cell wall for keeping the cellular integrity of Fungi [155,156]. It is also an essential element of nematode eggshell and pharynx [157] and the cuticles and gut lining of insects [158]. Chitinases are a diverse group of enzymes that catalyze the degradation of chitin by cleaving the β-(1–4) linkages, and some of them are also known to have antimicrobial activities [159]. The common buckwheat (*Fagopyrum esculentum* Moench) allergen Feg e 4 is known to be an antimicrobial peptide [160]. The structures of many chitinases have been determined, including that of corn allergen Zea m 8 with 3 disulfide bonds (PDB: 4MCK; Figure 1J).

**Thaumatin-like proteins.** Thaumatin-like proteins are also pathogenesis-related proteins known as PR-5 [134]. They are produced in response to a variety of stresses in many plants. Thaumatin purified from the katamfe (*Thaumatococcus daniellii*) berries taste 10^5^ times sweeter than sucrose on a molar base [161]. Known thaumatin-like food allergens include apple (*Malus domestica* Borkh) allergen Mal d 2, banana (*Musa acuminata* Colla) allergen Mus a 4, bell pepper (*Capsicum annuum* L.) allergen Cap a 1, cherry (*Prunus avium* (L.) L.) allergen Pru av 2, kiwi (*Actinidia deliciosa* (A.Chev.) C.F.Liang and A.R.Ferguson) allergen Act d 2, and peach (*Prunus persica* (L.) Batsch) allergen Pru p 2. The thaumatin-like proteins are also known as pollen allergens. Numerous structures of wild-type and mutant thaumatin-like proteins have been characterized [162], including banana allergen Mus a 4 (Figure 1K) [163].

**Figure 3:**
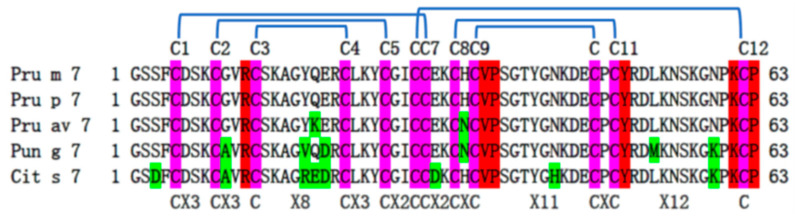
Sequence alignment of known GRP allergens. Cysteines are shown with a magenta background. The sequences of a large number of GRPs with a variable number of residues between the signal peptide and the first cysteine are also available (see text). Positions that tolerate amino acid changes are shown with a green background. Conserved residues in a sequence alignment included more GRPs are shown with a red background. The numbering of the cysteines and the disulfide bond connectivities are shown on top of the alignment. The sequences of the allergens were obtained following the allergen.org links and the alignment of the sequences was generated using Clustal Omega.

**Gibberellin-regulated proteins.** Six of the known food allergens belong to the gibberellin-regulated protein (GRP) family (GASA: PF02704). They are also known as Snakin, GASA, GRP, and peamaclein-like proteins. GRPs are ubiquitously expressed in monocots andeudicots. Many different GRPs have been identified in various plant species including rice [164], apple [165], and wheat [166]. GRPs play important roles in regulating different biological processes such as seed germination, root formation, stem elongation, flowering, etc. [167]. GRP genes are located on many different chromosomes in the genome of a plant [164,166], GRPs are small proteins expressed with a signal peptide, and the mature protein is localized to different parts of the cell including cell wall, chloroplast, vacuole, extracellular membrane, and mitochondria [164]. The main characteristic of the GRPs is their *C*-terminal 60-amino acid GASA domain which contains 12 conserved cysteines in a pattern of C1X_3_C2X_3_C3X_7–11_C4X_3_C5X_2_C6C7X_2_C8X_1–3_C9X_11_C10X_1–2_C11X_11–14_KC12P [168]. At the *N*-terminal of the mature GRPs is a variable region that is reported to contain 7–31 residues rich in polar amino acids [165,169]. Whether there is a correlation between the length of the *N*-terminal variable region of the GRPs and their localization to different plant organs, reproductive structures, or organelles of plant cells is not well studied. Besides the GRP food allergens, there are also GRP pollen allergens in the WHO/IUIS allergen database. The full sequences of these pollen allergens are currently not available, and all the GRP food allergens have a 4-residue *N*-terminal variable region in the mature proteins. The Clustal Omega [170] alignment of the sequences of these allergens and those of selected GRPs from other food is shown in Figure 3. A number of GRP proteins, including potato snakin1, have been demonstrated to have antimicrobial activities [171,172].

The crystal structure of potato snakin1 was recently determined by obtaining crystals of a racemic mixture of the L- and D-proteins obtained with total chemical synthesis [173]. The structure of the potato snakin1 and the disulfide bond connections of this antimicrobial protein is shown in Figure 1L. It is worth noting that C2X_3_C3X_7–11_C4X_3_C5 in the GRPs can be considered as a cC3C motif. Moreover, this cC3C has overlapping C3C on both sides of the motif, C1X_3_C2 on one side, and C5X_3_C7 on the other. Thus, C1, C2, C3, C4, C5, and C7 and the sequence gaps separating these cysteines constitute an extended cC3C. Because a full turn of an α-helix is 3.6 amino acids, a three-residue separation places the cysteines in a C3C on the same side of an α-helix. A cC3C provides two disulfide bonds to stabilize the two α-helixes connected by the loop constituted by the residues between the couple pair of C3Cs. An overlapping C3C (i.e., C3C3C) places three cysteines on the same side of an α-helix. The coupling of two overlapping C3Cs contributes three disulfide bonds for stabilizing the antiparallel α-helixes, as illustrated by the GRP food allergens.

## 3. IgE Epitopes

In sensitizing a predisposed individual, both the properties of the linear peptide sequence of an allergen and the properties of the allergen surface may play crucial roles [174]. In general, the majority of antibodies are specific to conformational epitopes [175,176]. However, IgE epitope studies have been focused nearly exclusively on linear epitopes. Earlier identification of linear IgE-binding epitopes was carried out by chemically synthesizing overlapping peptides spanning the whole sequence of an allergen and detecting the presence of IgE antibodies that recognize any of the peptides in dot blot experiments [177]. Recently, microarray technology has enabled the printing of a large number of peptides on chips for linear IgE epitope mapping [178,179]. So far, using sera from specific patient groups, dominant linear IgE epitopes in several allergens have been identified for selected allergens including those in milk [180,181,182], peanuts [68,183,184,185,186], brown shrimp [187], English walnuts [188], cashews [71,90], oyster [189], etc. Recently, we reported a method of recombinantly expressing overlapping peptides fused with human TL1A [190]. The peptide also contained a His tag that was used as a positive control for producing uniform spots. Incorporating a positive control in synthesizing the peptide spots or in printing the microarrays by adding a short epitope tag in future studies may result in better Information about dominant epitopes. Such information can be used to generate hypoallergic peptides and hypoallergens for the development of peptide immunotherapies and food allergen immunotherapies [191].

In contrast, hardly any information is available about conformational IgE epitopes of food allergens although such information is essential for fully understanding the molecular basis of protein allergenicity. To our knowledge, the structures of βLG in complex with the antigen-binding fragment (Fab) of a recombinant IgE [192], IgE-derived Fabs complex with pollen and contact allergens [193,194,195], and the FV region of a monoclonal IgE (raised against a 2,4-dinitrophenyl hapten) in complex with a peptide displayed on the active site loop of thioredoxin [196] are the only structures available for characterizing a conformational IgE epitope experimentally. Nevertheless, many investigations attempted to infer information about IgE epitopes by studying the co-structures of allergens and monoclonal IgG antibodies. Thus, currently, methodologies for determining dominant IgE epitopes, especially for conformational epitopes studies, seem to be inadequate to meet the research requirements.

The current wisdom attributes differences among individuals with different food allergies to the antibody–allergen interface. Patients with persistent allergies are believed to have IgE antibodies that recognize mainly linear epitopes, while individuals with transient allergies have IgE antibodies that recognize a higher proportion of conformational epitopes [197]. However, to our knowledge, there is no applicable method to map conformational epitopes recognized by the IgE repertoire in the serum of a patient. We believe there is a possibility that the current opinions about the importance of conformational epitopes in persistent allergies might be incorrect because currently no method can be used systematically map conformational epitopes.

## 4. Thermostability

For a food protein to sensitize a person or trigger reactions in a patient, the protein cannot be fully hydrolyzed in the gastrointestinal tract. The protein or smaller peptide pieces of it must survive the attack from digestive enzymes in the gastrointestinal tract and reach the immune system with intact IgE epitopes. The stability of food proteins may be one of the contributing factors to their allergenicity, and allergens from many important plant food allergen families are known to be stable in terms of heat resistance. The peach NsLTP allergen Pru p 3 was reported to maintain its secondary structures after being heated to 95 °C for 15 min at pH 3.4 [198]. The denaturation temperature of Brazil nut allergen Ber e 1 and peanut allergen Ara h 2 (both 2S seed storage proteins) exceeded 100 °C at neutral pH [58,199], and the thermal denaturation of Ber e 1 at low pH was fully reversible [199]. Peanut allergen Ara h 3, an 11S protein, was reported to denature at 92 °C [200], and pine nut allergen Pin k 2, a 7S protein, is also known to have a denaturation temperature over 93 °C [96]. The peach GRP allergen Pru p 7 was reported to maintain its native structure at 90 °C [201]. However, plant food allergens from other dominating families do not have high stability to resist thermal denaturation. The muskmelon allergen Cuc m 2, a profilin, has a reported melting temperature of about 56 °C [129], and celery allergen Api g 1, a PR-10, has a reported denaturation temperature between 70 and 73 °C [202]. Peanut oleosin allergens Ara h 10 and Ara h 11 were reported to have a denaturation midpoint lower than 60 °C [203], and the thermal unfolding of apple allergen Mal d 2 was reported to be reversible which happened at 70 °C [204]. Thus, the thermostability of plant food allergens varies considerably. It might be logical to consider stability to heat treatment as a contributing factor to the allergenicity of a protein. However, heating can modify some amino acids of an allergen, changing its IgE binding properties and altering the relative abundance of soluble allergens within a food [205,206,207]. Thermostability alone probably cannot be used reliably to predict if a protein would be an allergen.

## 5. Resistance to Digest

For the allergenicity assessment of genetically modified organisms with a newly introduced protein, regulatory agencies in different countries and regional organizations require the stability of the protein when treated with simulated gastric and intestinal fluid. In the literature, however, many different methods were used for studying the digestion stability of food proteins [208]. For example, to mimic the conditions in the stomach, simulated gastric digestion with pepsin was at different pH (1.2–2.5), and there is no standard for the duration of the reaction [199,209,210]. Furthermore, the digestion stabilities of different known allergens reported in a single study can be quite different when assessed parallel with the same protocol [211], and proteins with or without known to be allergenic were demonstrated to have similar stability to pepsin digestion [209,212]. The food matrix and food processing may also affect the digestibility of food proteins [213]. Although we were not aware of a systematic study investigating whether there is a difference between the abilities of allergens and other proteins of plant sources in surviving the digestive systems of allergic and control subjects, it is known that IgE may be present in the gut in food allergy patients [214], and a portion of two ingested food allergens of animal sources (Bos d 5, Gal d 2) is absorbed and transported throughout the body in an antibody-reactive form including through the gut of control subjects [215]. Intuitively, the digestibility may be an important contributor to the allergenicity of a food protein, but there is no standard method to characterize it and available data indicated that it alone cannot be used as a reliable indicator in identifying food allergens.

## 6. Cross-Reactivity

As noticed above, PR-10 and profilins, actin-associated proteins present in all eukaryotic cells, have been considered panallergens that may cause cross-reactions. The sequence similarities among the orthologous allergens in some other protein families can also be high, even those in species that are not close phylogenetic relatives. Significant cross-reactivity among legumes was recognized [216], and cross-reactivities between peanuts, a legume, and tree nuts were also frequently reported [217]. Specific IgE to different allergens has been reported to be most helpful in predicting patients’ cross-reactions to different foods. For example, omega-5 gliadin-specific IgE antibodies of wheat-allergic patients were reported to be a useful predictor for barley allergy [218]. Ara h 2 could contribute to the high incidence of peanut-allergic individuals’ sensitization to tree nuts [217]. However, though the co-sensitization between legumes in patient groups allergic to different legumes could be partially attributed to the 2S albumins, and the main allergens responsible for the co-sensitization in other studies were reported to be the 11S and 7S globulins [219]. In addition, IgE cross-reactivities were also reported between peanut allergens Ara h 1, Ara h 2, and Ara h 3 [220], but a recent study suggested the reported cross-reactivities likely resulted from the complex formation of the allergens [221]. Furthermore, IgE cross-reactivity between different foods was reported as not always being clinically relevant [219]. This is not surprising as IgE antibodies specific to more than one epitope of a monomeric allergen are required to cross-link IgE receptors to trigger allergic reactions. Further investigations are needed to provide better information about allergen cross-reactivity with patients’ IgE antibodies and their usage in predicting possible allergies. Nevertheless, thermostability, resistance to peptidase digestion, and sequence similarities are among the factors to consider for new proteins’ allergenicity. Although a lot of quality data have been published and experts’ opinions and regulatory guidelines updated, a clear roadmap for assessing the allergenicity and safety of new proteins is still an unachieved objective, and the challenges in accessing their safe use remain to be overcome [222].

## 7. Summary and Perspectives

In summary, information obtained from stability studies suggested that humans ingest folded, native allergens even if the food is conventionally processed. It appears that the importance of conformational epitopes in food allergens is currently underestimated due to the lack of information. Knowledge of more conformational epitopes is needed to understand better the structural determinants of protein allergenicity.

Many intrinsic characteristics of a food protein may be contributing factors for the protein to be allergenic. The amino acid sequences of proteins drive their three-dimensional structures. Proteins are separated into thoughts of families based on their sequence and structural features. Known food allergens can be grouped into families based on which protein families they belong to. The number of known allergen families is much smaller than the total number of protein families. Thus, the structure of a protein, including its shape and surface properties, may contribute to its allergenicity. Taken together, if a protein’s orthologs in many foods are known to be allergens, it may be suspected to be an allergen and warrant further investigation. On the other hand, recent identification of new food allergens belonging to protein families not known to have allergens before indicated that more research on food allergens are needed to better understand the possible relevance of the properties used for protein classification with the allergenicity of food proteins. In addition, further investigation targeting those protein families with only one allergen representative and assessing the allergenicity of the orthologous proteins with patient sera may also provide important information.

Because the determinants of linear IgE epitopes can be as short as eight amino acids, the sequence property of a short stretch of a peptide in the protein may also contribute to the allergenicity of the protein. Directly linked to the molecular structure of a protein is its thermostability which is also believed to be an important factor for protein allergenicity. Food allergens may also share the characteristics of being able to resist digestion. The abundance of a protein may make it more likely for a portion of it to survive proteolysis than other proteins with similar resistance to digestion. However, one cannot confidently predict the allergenic potential of a protein solely based on one of these factors.

## Figures and Tables

**Table 1:** Protein families with the most known food allergens from plant sources.

Protein Family ^a^	No. of Allergens	Proteins	No. of Allergens
PF00234: Tryp_alpha_amyl	74	Nsltp	42
		2S albumin	26
		Others	6
PF00190: Cupin_1	39	11S	19
		7S	20
PF00235: Profilin	27	Profilin	
PF00407: Bet_v_1	19	PR-10	
PF01277: Oleosin	10	Oleosin	
PF00187: Chitin_bind_1	7	Chitinases	
(PF00182: Glyco_hydro_19)			
PF00314: Thaumatin	6	Thaumatin-like	
PF02704: GASA	6	Gibberellin regulated protein	
PF04702: Vicilin_N	6	N-terminal of vicilin cC3C antimicrobial protein	
PF00112: Peptidase_C1			
PF08246: Inhibitor_I29	3	Cysteine protease	
PF00197: Kunitz_legume	3	Protease inhibitors	
PF00304: Gamma-thionin	3	Defensin	

^a^ The sequences of the allergen were obtained by following the links in the allergen.org database, and the protein families were determined by searching the Pfam/InterPro database (www.ebi.ac.uk/interpro, accessed on 14 April 2023).

## Data Availability

The data used to support the findings of this study can be made available by the corresponding author upon request.
